# Copper-Containing Amine Oxidases and FAD-Dependent Polyamine Oxidases Are Key Players in Plant Tissue Differentiation and Organ Development

**DOI:** 10.3389/fpls.2016.00824

**Published:** 2016-06-28

**Authors:** Paraskevi Tavladoraki, Alessandra Cona, Riccardo Angelini

**Affiliations:** Laboratory of Biochemistry, Physiology and Biotechnology of Plants, Department of Science, University “Roma Tre”Rome, Italy

**Keywords:** polyamines, copper amine oxidases, FAD-dependent polyamine oxidases, cell-wall, tissue differentiation, reactive oxygen species, growth regulation, programmed cell death

## Abstract

Plant polyamines are catabolized by two classes of amine oxidases, the copper amine oxidases (CuAOs) and the flavin adenine dinucleotide (FAD)-dependent polyamine oxidases (PAOs). These enzymes differ to each other in substrate specificity, catalytic mechanism and subcellular localization. CuAOs and PAOs contribute to several physiological processes both through the control of polyamine homeostasis and as sources of biologically-active reaction products. CuAOs and PAOs have been found at high level in the cell-wall of several species belonging to Fabaceae and Poaceae families, respectively, especially in tissues fated to undertake extensive wall loosening/stiffening events and/or in cells undergoing programmed cell death (PCD). Apoplastic CuAOs and PAOs have been shown to play a key role as a source of H_2_O_2_ in light- or developmentally-regulated differentiation events, thus influencing cell-wall architecture and maturation as well as PCD. Moreover, growing evidence suggests a key role of intracellular CuAOs and PAOs in several facets of plant development. Here, we discuss recent advances in understanding the contribution of different CuAOs/PAOs, as well as their cross-talk with different intracellular and apoplastic metabolic pathways, in tissue differentiation and organ development.

## Copper-containing amine oxidases and FAD-dependent polyamine oxidases: a complex network

**Figure 1:**
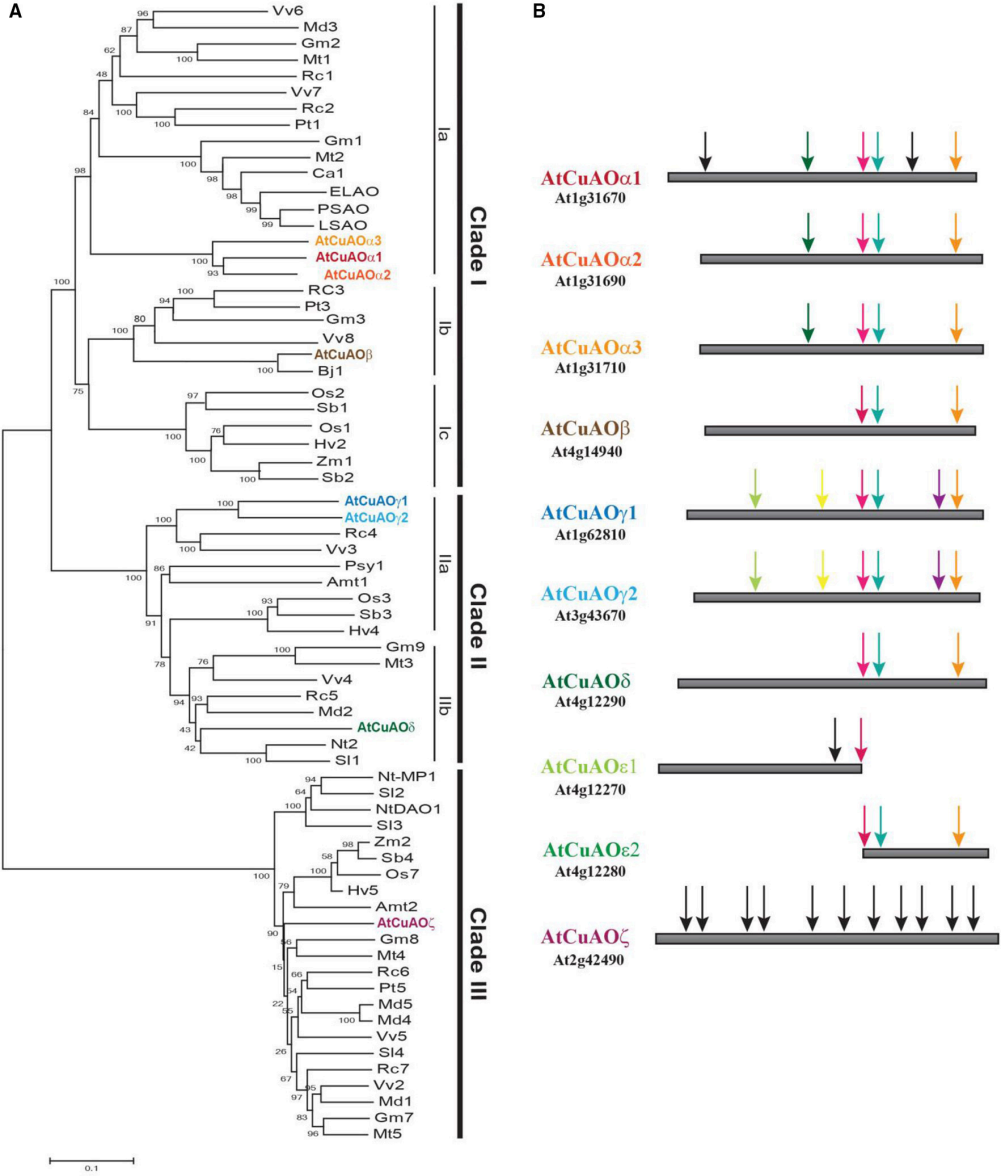
**Sequence analysis of plant CuAOs. (A)** Phylogenetic analysis of CuAOs from selected spermatophytes. Plant CuAOs form three principal supported clades (I-III). Clade I consists of three groups (groups a and b consisting of CuAOs from dicots and group c from monocots), the reciprocal relationship of which is not well resolved. For simplicity reasons, for each distinct group only a representative CuAO from each plant species was considered. Amino acid sequences were aligned with ClustalW (McWilliam et al., 2013) and phylogenetic analysis was performed using MEGA5 (Tamura et al., 2011) software with the neighbor-joining algorithm. Bootstrap values obtained with 1000 replicates are indicated at the nodes. Accession number of proteins are indicated in Supplementary Table S1. **(B)** Genomic sequence analysis of Arabidopsis *CuAOs* (*AtCuAOs*). Black arrows show not conserved intron positions, while arrows of the same color indicate conserved intron positions. All *AtCuAOs* of clades I and II have three introns at conserved positions which suggests a recent common ancestor. However, based on the presence of additional introns, some of them placed at positions conserved among the members of the same group, but not among those of different groups, an independent evolution of the *AtCuAOs* from the different groups can be suggested. *AtCuAO*ζ of clade III appears evolutionarily distant from *AtCuAOs* of clade I and II. Amt, *Amborella trichopoda*; Bj, *Brassica juncea*; Ca, *Cicer arietinum*; ELAO, CuAO from latex of *Euphorbia characias*; Gm, *Glycine max*; Hv, *Hordeum vulgare*; LSAO, CuAO from seedlings of *Lens culinaris*; Md, *Malus domestica*; Mt, *Medicago truncatula*; Nt, *Nicotiana tabacum*; Os, *Oryza sativa*; PSAO, CuAO from seedlings of *Pisum sativum*; Psy, *Pinus sylvestris*; Pt, *Populus trichocarpa*; Rc, *Ricinus communis*; Sb, *Sorghum bicolor*; Sl, *Solanum lycopersicum*; Vv, *Vitis vinifera*; Zm, *Zea mays*.

In plants, the polyamines (PAs) putrescine (Put), cadaverine (Cad), spermidine (Spd), spermine (Spm), and thermospermine (Therm-Spm) are involved in several physiological processes, such as cell proliferation, differentiation and defense responses (Takahashi and Kakehi, 2010; Marina et al., 2013; Jiménez-Bremont et al., 2014; Tiburcio et al., 2014; Pál et al., 2015; Strohm et al., 2015; Yoshimoto et al., 2016). PAs are oxidized by a heterogeneous class of enzymes which includes copper-containing amine oxidases (CuAOs) and FAD-dependent polyamine oxidases (PAOs) (Cona et al., 2006a; Angelini et al., 2010; Tavladoraki et al., 2012). CuAOs oxidize mainly Put and Cad, and less efficiently Spd and Spm at the primary amino groups, producing ammonia, H_2_O_2_ and an aminoaldehyde and are thus considered involved in PA terminal catabolism. In *Arabidopsis thaliana* 10 *CuAO* genes are present, among which only eight encode for putative functional CuAOs [*AtCuAO*α*1* (*At1g31670*); *AtCuAO*α*2* (*At1g31690*); *AtCuAO*α*3* (*At1g31710*; previously *AtCuAO2*); *AtCuAO*β (*At4g14940*; prev. *ATAO1* or *AtAO1*); *AtCuAO*γ*1* (*At1g62810*; prev. *AtCuAO1*); *AtCuAO*γ*2* (*At3g43670*); *AtCuAO*δ (At4g12290, prev. *AtCuAO*δ*2*); *AtCuAO*ζ (*At2g42490*; prev. *AtCuAO3* or *AtCuAO1*)]^1^ (Figure 1). The remaining two genes *AtCuAO*ε*1* (*At4g12270*; prev. *AtCuAO*ε) and *AtCuAO*ε*2* (*At4g12280*; prev. *AtCuAO*δ*1*) are consecutive fragments of a copy of *AtCuAO*δ gene. Phylogenetic analysis evidenced that plant CuAOs form three clades (I-III), clade I being composed of three subgroups (Ia-Ic) and clade II of two (IIa and IIb; Figure 1A). Furthermore, genomic sequence analysis demonstrated that the Arabidopsis CuAOs of clades I and II, but not of clade III, present a similar gene structure to each other with three introns at conserved positions (Figure 1B). This suggests that *AtCuAO*α*1* to *AtCuAO*δ are recent derivatives of a common ancestor.

AtCuAOβ (clade Ic), AtCuAOγ1 (clade IIa) as well as *Pisum sativum, Lens culinaris* and *Euphorbia characias* CuAOs (PSAO, LSAO, and ELAO, respectively; clade Ia) are localized in the apoplast (Rossi et al., 1992; Tipping and McPherson, 1995; Møller and McPherson, 1998; Padiglia et al., 2002; Boudart et al., 2005; Planas-Portell et al., 2013), whereas AtCuAOζ, *Malus domestica* CuAO1 (MdAO1) and the other members of clade III in peroxisomes (Planas-Portell et al., 2013; Naconsie et al., 2014; Qu et al., 2014; Zarei et al., 2015a; Table 1). Peroxisomal localization was also shown for AtCuAOα3 (Planas-Portell et al., 2013) despite the apparent lack of canonical signal for peroxisomal localization and the fact that it is clustered together with the extracellular PSAO and LSAO. For MdAO2, which is clustered together with AtCuAOδ in clade IIb, both intracellular and apoplastic localization was shown (Zarei et al., 2015a).

**Table 1:** **Characteristics and functions of plant CuAOs and PAOs**.

		**Localization**	**Substrate Preference**	**Function**	**References**
**COPPER AMINE OXIDASES**					
Clade la	AtCuAOα1^a^	–	–	–	–
	AtCuAOα2	–	–	–	–
	AtCuAOα3	Peroxisomes	Put, Spd	–	Planas-Portell et al., 2013
	PSAO	Apoplast	Put, Spd, Spm	–	Tipping and McPherson, 1995; Moschou et al., 2012
	ELAO	Apoplast	Put, Benzylamine, Tyramine	–	Pintus et al., 2013
	LSAO	Apoplast	Put, Spd, Spm, Tryptamine	–	Rossi et al., 1992; Medda et al., 1997; Tavladoraki et al., 2012
	CaCuAO	Apoplast	Put	Wound healing, defense response	Rea et al., 2002
Clade Ib	AtCuAOβ	Apoplast	Put, Spd	Vascular development	Møller and McPherson, 1998; Ghuge et al., 2015a
Clade IIa	AtCuAOγ1	Apoplast	Put, Spd	PA- and ABA-mediated NO production	Wimalasekera et al., 2011; Planas-Portell et al., 2013
	AtCuAOγ2	–	–	–	–
Clade IIb	AtCuAOδ	–	–	–	–
	MdAO2	Apoplast	2-Phenylethylamine, Tyramine, Ethanolamine, Ethylamine, Tryptamine	Fruit flavor, flower fragance	Zarei et al., 2015a
Clade III	AtCuAOζ		Put, Spd, *N*-methyl-Put, Cad	ABA-induced stomatal closure	Planas-Portell et al., 2013; Naconsie et al., 2014; Qu et al., 2014
	NtDAO1	Peroxisomes	Put, *N*-methyl-Put, Cad	–	Naconsie et al., 2014
	MdAO1		Dap, Put, Cad	–	Zarei et al., 2015a
	Nt-MPO1		*N*-methyl-Put, *N*-methyl-Dap, Put, Dap, Cad	Alkaloid synthesis	Katoh et al., 2007; Naconsie et al., 2014
**POLYAMINE OXIDASES**					
Clade I	AtPAO1	Cytosol	Nor-Spm, Therm-Spm, Spm	Stress response	Tavladoraki et al., 2006; Takahashi et al., 2010; Sagor et al., 2016
	GhPAO1		Spm	Defense response, differentiation of embryogenic callus	Cheng et al., 2015; Mo et al., 2015
Clade II	ZmPAO1	Apoplast	Spd, Spm	Cell wall differentiation	Cona et al., 2006a
	HvPAO1	Apoplast	–	–	Cervelli et al., 2001; Cona et al., 2006a
	HvPAO2	Vacuole	Spm, Spd	–	Cervelli et al., 2001; Cona et al., 2006a
	OsPAO7	Apoplast	Spm, Spd, *N*^1^-acetyl-Spm	–	Liu et al., 2014b
Clade III	AtPAO5		Spm, Therm-Spm, Nor-Spm, *N*^1^-acetyl-Spm	Polyamine homeostasis, plant growth, stress response	Ahou et al., 2014; Kim et al., 2014; Zarza et al., 2016
	SelPAO5		Therm-Spm, Spm, Nor-Spm, *N*^1^-acetyl-Spm	–	Sagor et al., 2015
	OsPAO1	Cytosol	Spm, Therm-Spm, Nor-Spm, *N*^1^-acetyl-Spm	Plant growth	Liu et al., 2014a,c
	BjPAO1		–	Shoot regeneration	Lim et al., 2006
	GhPAO4		–	Differentiation of embryogenic callus	Cheng et al., 2015
Clade IV	AtPAO2		Spm, Spd, Nor-Spm	Stress response	Moschou et al., 2008b; Takahashi et al., 2010; Fincato et al., 2011; Wimalasekera et al., 2015; Sagor et al., 2016
	AtPAO3	Peroxisomes	Spd, Spm, Nor-Spm	Pollen tip growth	Moschou et al., 2008b; Takahashi et al., 2010; Fincato et al., 2011; Wu et al., 2010
	AtPAO4		Spm	Senescence	Moschou et al., 2008b; Kamada-Nobusada et al., 2008; Takahashi et al., 2010; Fincato et al., 2011; Sequera-Mutiozabal et al., 2016
	OsPAO3		Spd, Nor-Spm	–	Ono et al., 2012
	OsPAO4		Spm, Therm-Spm, Nor-Spm	–	Ono et al., 2012
	OsPAO5		Spm, Therm-Spm, Nor-Spm	–	Ono et al., 2012

a *At, Arabidopsis thaliana; Bj, Brassica juncea; Ca, Cicer arietinum; Gh, Gossypium hirsutum; Hv, Hordeum vulgare; Md, Malus domestica; Nt, Nicotiana tabacum; Os, Oryza sativa; Sel, Selaginella lepidophylla; SI, Solanum lycopersicum; Zm, Zea mays. ELAO, LSAO, and PSAO: CuAO from Euphorbia characias latex. Lens culinaris seedlings and Pisum sativum seedlings, respectively*.

Although, most of the so far characterized CuAOs, such as AtCuAOβ, AtCuAOγ1, AtCuAOα3, AtCuAOζ, PSAO, LSAO, and *Nicotiana tabacum* CuAO1 (NtDAO1), oxidize mainly Put, Cad, and Spd (Rossi et al., 1992; Tipping and McPherson, 1995; Møller and McPherson, 1998; Planas-Portell et al., 2013; Naconsie et al., 2014), MdAO1 of clade III shows preference for 1,3-diaminopropane (Dap), having no activity with Spd (Zarei et al., 2015a). Furthermore, AtCuAOζ and NtDAO1 oxidize also *N*-methyl-Put, though less efficiently than the non-methylated diamine. Thus, they differ from the *N. tabacum N*-methylputrescine oxidase (Nt-MPO1), which shows preference for *N*-methyl-Put and is involved in nicotine biosynthesis, although all three proteins are clustered together in clade III (Heim et al., 2007; Katoh et al., 2007; Dewey and Xie, 2013; Naconsie et al., 2014). This indicates that clade III consists of a heterogeneous group of CuAOs. Another remarkable finding is the higher catalytic activity of MdAO2 with monoamines, such as 2-phenylethylamine, tyramine and tryptamine, than with Put and Spd (Zarei et al., 2015a). Interestingly, tyramine is also a substrate of ELAO whereas tryptamine and other indoleamines are both substrates and inhibitors of LSAO (Medda et al., 1997; Pintus et al., 2013). It was speculated that 2-phenylacetaldehyde produced by MdAO2-mediated oxidation of 2-phenylethylamine may be converted in fruits to 2-phenylethanol, a volatile compound that is a major contributor to fruit flavor and flower fragrance. It is also possible that 4-hydroxyphenylacetaldehyde produced by tyramine oxidation is involved in benzylisoquinoline alkaloid biosynthesis in plants (Zarei et al., 2015a).

PAOs oxidize the secondary amino groups of a series of PAs and reaction products depend on the catalytic mechanism and substrate specificity. The apoplastic PAOs oxidize the carbon at the *endo*-side of the *N*^4^ atom of Spd and Spm producing Dap, H_2_O_2_, and an aminoaldehyde (Tavladoraki et al., 1998; Cervelli et al., 2001; Liu et al., 2014b), whereas all the intracellular PAOs oxidize the carbon at the *exo*-side of the *N*^4^ atom of Spd or Spm, to produce Put or Spd, respectively, together with H_2_O_2_ and 3-aminopropanal (Tavladoraki et al., 2006; Kamada-Nobusada et al., 2008; Moschou et al., 2008b; Fincato et al., 2011; Ahou et al., 2014; Kim et al., 2014; Liu et al., 2014a; Mo et al., 2015). Some of the intracellular PAOs are also able to oxidize Therm-Spm and norspermine (Nor-Spm) with the production of Spd and norspermidine (Nor-Spd), respectively (Tavladoraki et al., 2006; Fincato et al., 2011; Kim et al., 2014; Liu et al., 2014a). Recently, a *Selaginella lepidophylla* PAO (SelPAO5) was shown to produce Nor-Spd from Therm-Spm (Sagor et al., 2015). These differences in reaction products reflect differences in position and orientation of the substrate inside the catalytic site. Both the *exo*- and *endo*-mode of PA oxidation produce a biologically active diamine or triamine which can be converted to higher PAs. Indeed, even Dap, which has a role in the control of stomata movement through its acetylated form (Jammes et al., 2014), can be converted by aminopropyltransferases to Nor-Spd and subsequently to Nor-Spm, two PAs correlated to stress tolerance (Kuehn et al., 1990; Fuell et al., 2010; Sagor et al., 2015). In this way, all PAOs can be considered involved in PA back-conversion. This view changes the prevailing idea that the PAOs with an *endo*-mode of substrate cleavage are involved in PA terminal catabolism, thus attributing to CuAOs the role of PA terminal catabolism which permits nitrogen and carbon re-assimilation to various biochemical reactions (Moschou et al., 2012).

In Arabidopsis five PAOs are present (AtPAO1-AtPAO5), which are localized intracellularly and show an *exo*-mode of substrate oxidation. AtPAO1 and AtPAO5 present cytosolic localization and a preference for Spm, Therm-Spm, and Nor-Spm, as substrates (Tavladoraki et al., 2006; Ahou et al., 2014; Kim et al., 2014; Liu et al., 2014a). AtPAO5 accepts also *N*^1^-acetyl-Spm as a substrate and appears to be a peculiar PAO, having a better activity as a dehydrogenase rather than as an oxidase (Ahou et al., 2014). AtPAO2, AtPAO3, and AtPAO4 are localized in the peroxisomes and oxidize both Spd and Spm (Kamada-Nobusada et al., 2008; Moschou et al., 2008b; Takahashi et al., 2010; Fincato et al., 2011; Sequera-Mutiozabal et al., 2016). Furthermore, AtPAO2, AtPAO3, and AtPAO4 present similar gene structures and tissue-specific expression patterns (root tips, guard cells and pollen grains; Takahashi et al., 2010; Fincato et al., 2012). Phylogenetic studies showed that PAOs are divided into four major clades (Liu et al., 2014a; Wang and Liu, 2015). Worth noticing is the fact that the PAOs of the same clade present some common characteristics (Table 1). In particular, clade I PAOs have cytosolic localization and oxidize specifically Spm but not Spd, while clade IV PAOs present peroxisomal localization and specificity for either Spm or Spd or both. The apoplastic PAOs of clade II show preference for both Spm and Spd and are characterized by a *k*_*cat*_ value at least 10-fold higher than that of the PAOs of the other clades. Clade III PAOs are cytosolic enzymes which recognize Spm, Therm-Spm, Nor-Spm, and *N*^1^-acetyl Spm as substrates and are regulated by PAs (Ahou et al., 2014; Kim et al., 2014; Liu et al., 2014a; Wang and Liu, 2015). Furthermore, they present very low *k*_*cat*_ values as oxidases, which indicate that not only AtPAO5 but all AtPAO5 orthologs may have activity mainly as dehydrogenases (Ahou et al., 2014).

## Cell-wall amine oxidases: driving ROS production in the apoplastic “hub”

Compelling evidence supports a key role for reactive oxygen species (ROS; superoxide anion, ${\text{O}}_{2}^{•-}$; H_2_O_2_; hydroxyl radical, ^•^OH; singlet oxygen, ^1^O_2_) and nitric oxide (NO) in orchestrating developmental processes, as well as in being involved in signaling of both local and systemic defense responses in plants. The apoplast is a major “hub” for these chemical species. Their accumulation in large amounts and the complexity of the regulatory mechanisms involved in their biosynthesis reflect the peculiar role of this compartment in physiological events that depend on temporarily regulated and spatially restricted ROS and NO signatures (Kärkönen and Kuchitsu, 2015). Indeed, ROS are key players in cell-wall loosening and stiffening, as well as in developmental cell death, and stress-related events, such as the two-phase oxidative burst, wound-healing and the hypersensitive response (De Pinto et al., 2012; Kärkönen and Kuchitsu, 2015). Furthermore, ROS generated in the apoplast may well influence Ca^2+^ transport across plasma membrane thus inducing a multiplicity of Ca^2+^-mediated responses (Gilroy et al., 2014; Pottosin et al., 2014). Systems involved in ROS biosynthesis in the apoplast include plasma membrane NADPH oxidases and quinone reductases, cell-wall peroxidases (PODs), oxalate oxidases, amine oxidases (Kärkönen and Kuchitsu, 2015). Non enzymatic ^•^OH production can be also driven by Fenton-type reaction dependent on a reducing agent (for example ascorbate), transition metal ions and H_2_O_2_ (Schopfer et al., 2002; Müller et al., 2009). Although, a very significative amount of data suggests NADPH oxidases and/or PODs to have a prevalent role in ROS production in response to microbial pathogens, wounding, as well as in development-related events, the contribution of other systems has been largely underestimated (Cona et al., 2006a,b; Monshausen et al., 2007; Angelini et al., 2008; Kärkönen and Kuchitsu, 2015; Roach et al., 2015). This consideration derives mainly from the analysis of literature data based on extensive use of diphenyleneiodonium (DPI), sodium azide or potassium cyanide and diethyldithiocarbamate (DDC) as diagnostic tools for the involvement of NADPH oxidase, POD or superoxide dismutase activity, respectively, in ROS production. However, these compounds are inhibitors of CuAOs (DDC, azide, cyanide) and PAOs (DPI) as well (Cona et al., 2006b; Medda et al., 2009). Noteworthy, a cross-talk has been recently suggested between AtPAO3 and NADPH oxidase activity, affecting ROS homeostasis and respiration rate in Arabidopsis (Andronis et al., 2014). On the other hand, several evidences support a role of PAs not only in ROS production, but also in that of NO (Tun et al., 2006; Pál et al., 2015). Indeed, loss-of-function *atcuao*γ*1* and *atpao2* mutants are impaired in PA- and/or abscisic acid (ABA)-mediated NO production (Wimalasekera et al., 2011, 2015).

## Polyamine oxidation in the apoplast: cell-wall modifications and growth regulation

Early studies in legumes, as well as in maize and tobacco plants evidenced a close correlation between tissue distribution of cell-wall associated CuAO, PAO, and POD activities (Federico and Angelini, 1991; Rea et al., 2002; Paschalidis and Roubelakis-Angelakis, 2005). These studies, also supported by the use of mechanism-based specific inhibitors, suggested a role of PA oxidation in providing H_2_O_2_ for POD activity during cell-wall cross-linking, lignification and/or suberization processes taking place in the course of organ development, de-etiolation, or wound-healing (Cona et al., 2003, 2006a, 2014; Angelini et al., 2008). A role for lignin synthesis in anther cell-walls has been also hypothesized for an *Oryza sativa* PAO (OsPAO7; Liu et al., 2014b). The major concern raised by these studies comes from the low levels or even absence of PAs in the apoplast under physiological growth conditions (Rea et al., 2004). However, this issue was overcome by the observation that PAs are secreted in the apoplastic milieu during specific developmental phases or under biotic and abiotic stress conditions (Yoda et al., 2003; Moschou et al., 2008a; Rodríguez et al., 2009). On the other hand, in *Zea mays*, PAO-mediated H_2_O_2_ production in the apoplast significantly contributes to leaf blade elongation, possibly through Fenton and/or Haber-Weiss type reactions driving ^•^OH synthesis (Rodríguez et al., 2009). A similar role has been suggested in *Glycine max* hypocotyls for the putative apoplastic GmCuAO1 (Delis et al., 2006). This contribution is particularly important under salt stress conditions which inhibit both NADPH oxidases and apoplastic POD activities and induce secretion of PAs in the apoplast (Rodríguez et al., 2009; Campestre et al., 2011; Shoresh et al., 2011).

## Apoplastic CuAOs and PAOs: developmental PCD and root xylem differentiation

It is well established that ROS have a key role in programmed cell death (PCD) in plants (De Pinto et al., 2012). Although, cell-wall maturation and lignification is intimately connected to the cell-death phase of xylem tissue differentiation, it is difficult to distinguish specific ROS contribution to each specific event (Bollhöner et al., 2012). Notably, transition between cell proliferation and tissue differentiation in the root is regulated independently by either hormonal balance (auxin and citokinin) or ROS (${\text{O}}_{2}^{•-}$ and H_2_O_2_) distribution (Tsukagoshi et al., 2010). Several lines of evidence links PA oxidation in the apoplast and concomitant ROS production with cell-wall maturation and developmental PCD in particular during xylem tissue differentiation. Møller and McPherson (1998) demonstrated that *AtCuAO*β expression in root xylem tissues precedes and overlaps with lignin synthesis in Arabidopsis. Furthermore, although the roots of loss-of-function *atcuao*β mutants did not display evident changes as compared to wild-type plants during normal growth, methyl jasmonate induced early protoxylem differentiation in roots of wild-type plants, concomitantly with increased *AtCuAO*β expression levels, decreased Put levels and H_2_O_2_ accumulation. As these events do not occur in *atcuao*β roots, a role of *AtCuAO*β in protoxylem differentiation under stress condition can be suggested (Ghuge et al., 2015a,c). On the other hand, tobacco plants over-expressing a fungal endopolygalacturonase, releasing cell-wall derived oligogalacturonides and mediating both developmental events and defense responses, showed higher CuAO activity, lower Put level, H_2_O_2_ accumulation and an earlier root xylem differentiation. These events were reverted by 2-bromoethylamine, a mechanism-based CuAO inhibitor (Cona et al., 2014). Furthermore, it has been observed that active ZmPAO and H_2_O_2_ accumulate in root xylem and xylem parenchyma tissues early during their differentiation (Tisi et al., 2011). *N*-prenylagmatine, a specific PAO inhibitor, inhibited secondary cell-wall deposition, while exogenous Spd induced DNA fragmentation and nuclei condensation, thus suggesting a role for PAO in providing H_2_O_2_ during secondary wall deposition and developmental PCD in xylem tissue (Tisi et al., 2011). Moreover, over-expression of *ZmPAO1*, as well as down-regulation of the gene encoding S-adenosyl-L-methionine decarboxylase via RNAi in tobacco plants promoted vascular cell differentiation and induced PCD in root cap cells suggesting that the balance between intracellular PA anabolism and apoplastic catabolism is an integrated signaling system coordinating PCD or stress tolerance (Moschou et al., 2008a; Tisi et al., 2011).

## Peroxisomal PAOs: involvement in pollen tube and root growth

PAs and ROS deriving from their oxidation regulate ion channels both in animals and plants during different physiological and stress-response processes directly influencing plasma membrane ion transport and/or acting as second messengers (Pegg, 2014; Pottosin et al., 2014). In particular, H_2_O_2_ produced through Spd oxidation by the peroxisomal AtPAO3, which is highly expressed in pollen grains also during pollen tube growth (Fincato et al., 2012), was shown to trigger the opening of hyperpolarization activated Ca^2+^-permeable channels in pollen tubes, thus altering the tip-specific cytosolic Ca^2+^ gradient which plays a pivotal role in controlling pollen tube elongation (Wu et al., 2010). Indeed, two loss-of-function Arabidopsis *atpao3* mutants presented reduced pollen tube growth rate and seed production. Peroxisomal OsPAO3, OsPAO4, and OsPAO5 (Ono et al., 2012) are coherently expressed in anthers (Liu et al., 2014b). More studies are necessary to determine in detail the effect of PAs on pollen maturation, since the effect seems to be complex involving PA conjugation, ROS formation/scavenging, and cell-death events (Aloisi et al., 2015). Furthermore, based on the vigorous root growth of *AtPAO2* over-expressor plants and the hypersensitivity of *atpao2* loss-of-function mutant plants to ABA, it has been recently hypothesized a positive function of *AtPAO2* in Arabidopsis root growth (Wimalasekera et al., 2015).

## CuAOs and PAOs in stomata movement and fruit ripening

ROS and calcium signatures in guard cells, as well as ion transport from the apoplast into the cytosol and from the cytosol for storage in the vacuole are important components of the regulatory network controlling stomata movements (Kollist et al., 2014; Murata et al., 2015). In *Vicia faba*, it has been shown that ABA-mediated stomata closure involves induction of an apoplastic CuAO activity as a source of H_2_O_2_, and that this activity is necessary to increase cytosolic Ca^2+^ levels in response to ABA (An et al., 2008). Consistently with these observations, the apoplastic AtCuAOβ was shown to be expressed in guard cells (Ghuge et al., 2015b). In addition, the peroxisomal AtCuAOζ which is expressed in guard cells is also involved in the ABA-mediated control of stomata opening (Qu et al., 2014). PAOs were also shown to contribute to the control of stomata movement in *Vitis vinifera* and *Arabidopsis* (Paschalidis et al., 2010; Hou et al., 2013).

PA catabolism has been also associated with grape and tomato fruit ripening (Agudelo-Romero et al., 2013; Tsaniklidis et al., 2016). Despite the increase of arginine decarboxylase expression levels during grape fruit ripening, the level of free and conjugated PAs was strongly decreased. This decrease was accompanied by up-regulation of two *CuAOs* and three *PAO* genes, one *CuAO* and two *PAOs* of them encoding for proteins with putative peroxisomal localization (Agudelo-Romero et al., 2013). Increase of CuAO and PAO activity, as well of H_2_O_2_ production during fruit maturation, was also observed. Furthermore, the relatively high expression levels of *Solanum lycopersicum* CuAO (SlCuAO1), which is clustered together with AtCuAOδ in clade IIb, was attributed to the implication of PA metabolism in physiological processes taking place during fruit ripening (Tsaniklidis et al., 2016). The up-regulation of CuAOs/PAOs during ripening may constitute a source of ROS for signaling events leading to the acceleration of the ripening process. It is also possible that PA catabolism interferes with plant hormonal pathways, such as ethylene and ABA (Agudelo-Romero et al., 2013). Alternatively, 4-aminobutanal produced through PA catabolism can be metabolized to γ-aminobutyric acid by an aminoaldehyde dehydrogenase (Zarei et al., 2015b) and enter into the Krebs cycle, thus constituting a link between nitrogen and carbon metabolism (Moschou et al., 2012).

## Cytosolic PAOs in the control of Therm-Spm levels

Loss-of-function Arabidopsis mutants for *AtPAO5* present increased levels of Therm-Spm, *N*^1^-acetyl-Spm and Spm which are the substrates of the enzyme (Ahou et al., 2014; Kim et al., 2014; Sagor et al., 2016; Zarza et al., 2016). Interestingly, the expression levels of *AtPAO5*, as well as of its functional orthologue in rice (*OsPAO1*), are controlled by Therm-Spm and Spm (Ahou et al., 2014; Liu et al., 2014a,c). This indicates the necessity for a fine tune regulation of PA levels for proper growth and stress response, since levels above an upper limit or below a threshold may be detrimental for the plants. Indeed, the *atpao5* mutants present altered growth parameters at late developmental stages mainly due to the increased Therm-Spm levels (Kim et al., 2014), as have been also observed in mutants for *Therm-Spm synthase* and *S-adenosylmethionine decarboxylase* with reduced Therm-Spm levels (Clay and Nelson, 2005; Ge et al., 2006; Yoshimoto et al., 2016). Furthermore, the *atpao5* mutants were hypersensitive to low doses of exogenous Therm-Spm (Kim et al., 2014; Liu et al., 2014c).

## Intracellular PAOs and somatic embryogenesis

Some studies suggest that in *Gossypium hirsutum* an AtPAO1-like (GhPAO1) and an AtPAO5-like (GhPAO4) PAO may play a crucial role in the generation and differentiation of embryogenic callus during somatic embryogenesis (Cheng et al., 2015). Indeed, PAO activity levels significantly increased during conversion of embryogenic callus into somatic embryos, and inhibition of PAO activity by 1,8-diaminooctane resulted in brown and necrotic cultures, and a significant decrease in both fresh weight and somatic embryo number. Importantly, the negative effects of 1,8-diaminooctane were reversed by application of exogenous H_2_O_2_. Furthermore, in Arabidopsis, *AtPAO5* and its *B. juncea* ortholog (*BjPAO*) have a role in shoot regeneration from root cultures (Lim et al., 2006).

## Concluding remarks

Numerous recent studies have evidenced an extraordinary complexity in *CuAO* and *PAO* gene families regarding catalytic activity, subcellular localization, expression pattern and physiological roles of the encoded proteins. Indeed, important links to developmental and stress-related events are emerging for CuAOs and PAOs through ROS/NO production and regulation of specific PA levels.

## Author contributions

All authors listed, have made substantial, direct and intellectual contribution to the work, and approved it for publication.

### Conflict of interest statement

The authors declare that the research was conducted in the absence of any commercial or financial relationships that could be construed as a potential conflict of interest.

## Footnotes

^1^Due to heterogeneity of *CuAO* gene classification in literature, we have renumbered them according to Qu et al. (2014) with some modifications taking into consideration the phylogenetic analysis (Figure 1). We trust that this will help for future use.
