# Supplementary material for: Copper-Containing Amine Oxidases and FAD-Dependent Polyamine Oxidases Are Key Players in Plant Tissue Differentiation and Organ Development
Source: Front Plant Sci. 2016 Jun 28;7:824. doi: 10.3389/fpls.2016.00824 (PMC4923165; doi:10.3389/fpls.2016.00824)
Supplement: Supplementary file 1 [file Table1.PDF]

Table S1. Species of origin and accession numbers of the CuAOs used in the phylogenetic analysis

Acronym	Species	Accession number	Acronym	Species	Accession number
**Amt1**	*Amborella trichopoda*	XP_006857816.2	**Nt-MP1**	*Nicotiana tabacum*	XP_009778427.1
**Amt2**	*Amborella trichopoda*	XP_006845257.1	**Nt2**	*Nicotiana tabacum*	AIE54293.1
**Atα1**	*Arabidopsis thaliana*	At1g31670	**Os1**	*Oryza sativa*	EEC82302.1
**Atα2**	*Arabidopsis thaliana*	At1g31690	**Os2**	*Oryza sativa*	EEC80539.1
**Atα3**	*Arabidopsis thaliana*	At1g31710	**Os3**	*Oryza sativa*	CAD39884.2
**Atβ**	*Arabidopsis thaliana*	At4g14940	**Os7**	*Oryza sativa*	EAZ31082.1
**Atγ1**	*Arabidopsis thaliana*	At1g62810	**PSAO**	*Pisum sativum*	Q43077.1
**Atγ2**	*Arabidopsis thaliana*	At3g43670	**Psy1**	*Pinus sylvestris*	ADQ37305.1
**Atδ**	*Arabidopsis thaliana*	At4g12290	**Pt1**	*Populus trichocarpa*	XP_002312527.2
**Atε1**	*Arabidopsis thaliana*	At4g12270	**Pt3**	*Populus trichocarpa*	XP_002314704.2
**Atε2**	*Arabidopsis thaliana*	At4g12280	**Pt5**	*Populus trichocarpa*	XP_002322194.2
**Bj1**	*Brassica juncea*	AAL47166.1	**Rc3**	*Ricinus communis*	XP_002516781.1
**Ca1**	*Cicer arietinum*	NP_001265996.1	**Rc2**	*Ricinus communis*	EEF45396.1
**EL**	*Euphorbia characias*	AF171698.2	**Rc1**	*Ricinus communis*	XP_002516777.1
**Gh**	*Gossypium hirsutum*	AGO02008.1	**Rc4**	*Ricinus communis*	XP_002509597.1
**Gm1**	*Glycine max*	NP_001237211.1	**Rc5**	*Ricinus communis*	XP_002509596.1
**Gm2**	*Glycine max*	XP_003556043.1	**Rc6**	*Ricinus communis*	XP_002511334.1
**Gm3**	*Glycine max*	KRH35530.1	**Rc7**	*Ricinus communis*	XP_002527922.1
**Gm7**	*Glycine max*	XP_003551224.1	**Sb1**	*Sorghum bicolor*	XP_002452575.1
**Gm8**	*Glycine max*	XP_003550715.1	**Sb2**	*Sorghum bicolor*	XP_002460889.1
**Gm9**	*Glycine max*	XP_003546898.1	**Sb3**	*Sorghum bicolor*	XP_002446210.1
**Hv2**	*Hordeum vulgare*	BAJ94038.1	**Sb4**	*Sorghum bicolor*	XP_002448036.1
**Hv4**	*Hordeum vulgare*	BAJ85075.1	**Sl1**	*Solanum lycopersicum*	NP_001296994.1
**Hv5**	*Hordeum vulgare*	EMS63127.1	**Sl2**	*Solanum lycopersicum*	XP_004244763.1
**LSAO**	*Lens culinaris*	P49252.3	**Sl3**	*Solanum lycopersicum*	XP_010322539.1
**Md1**	*Malus domestica*	AIS23644.1	**Sl4**	*Solanum lycopersicum*	XP_004239124.1
**Md2**	*Malus domestica*	AIS23645.1	**Vv8**	*Vitis vinifera*	CBI26238.3
**Md3**	*Malus domestica*	AIS23646.1	**Vv6**	*Vitis vinifera*	XP_002278244.1
**Md4**	*Malus domestica*	AIS23647.1	**Vv7**	*Vitis vinifera*	XP_002278327.1
**Md5**	*Malus domestica*	AIS23648.1	**Vv3**	*Vitis vinifera*	XP_003635614.2
**Mt1**	*Medicago truncatula*	XP_003592404.1	**Vv4**	*Vitis vinifera*	CBI34761.3
**Mt2**	*Medicago truncatula*	XP_013455559.1	**Vv2**	*Vitis vinifera*	XP_002273532.2
**Mt3**	*Medicago truncatula*	XP_003601419.1	**Vv5**	*Vitis vinifera*	XP_002277961.1
**Mt4**	*Medicago truncatula*	XP_003601195.1	**Zm1**	*Zea mays*	NP_001145964.1
**Mt5**	*Medicago truncatula*	XP_003613133.2	**Zm2**	*Zea mays*	NP_001169559.1
**NtDAO1**	*Nicotiana tabacum*	BAF49520.1			
